# COVID-19 as a Contributing Factor in the Development of Volkmann Ischemic Contracture: A Case Report

**DOI:** 10.7759/cureus.97573

**Published:** 2025-11-23

**Authors:** Tomas Maciulaitis, Patricija Glovackaite, Mindaugas Minderis

**Affiliations:** 1 Faculty of Medicine, Institute of Clinical Medicine, Clinic of Rheumatology, Orthopedics, Traumatology and Reconstructive Surgery, Vilnius University, Vilnius, LTU; 2 Faculty of Medicine, Vilnius University, Vilnius, LTU; 3 Center of Plastic and Reconstructive Surgery, Vilnius University Hospital Santaros Klinikos, Vilnius, LTU

**Keywords:** compartment syndrome, covid-19, neuropathy, pronator syndrome, volkmann ischemic contracture

## Abstract

Volkmann ischemic contracture (VIC) is a rare but devastating complication of untreated acute compartment syndrome, typically resulting in irreversible flexion deformities due to ischemic necrosis. While its pathophysiology is well understood, evolving clinical contexts, such as COVID-19, may present novel challenges in the early recognition and management of the condition.

We describe the case of a 55-year-old man who developed fixed flexion deformity involving the forearm flexor muscles following a misdiagnosis of compartment syndrome during COVID-19 isolation. Symptoms were initially attributed to cellulitis, and multiple superficial incisions were performed for presumed abscess drainage without confirmation of purulence or infection. Delayed intervention led to muscle necrosis and subsequent contracture. Upon tertiary referral, a diagnosis of VIC was established based on clinical findings. Surgical decompression, debridement, and neurolysis were performed, resulting in partial functional recovery.

This case highlights the importance of recognizing compartment syndrome, even in the absence of trauma, and raises the possibility that COVID-19 may contribute to the pathophysiology of VIC. We discuss mechanisms that include cytokine-mediated edema, endothelial dysfunction, diagnostic delay, and impaired wound healing.

## Introduction

Volkmann ischemic contracture (VIC) is a rare and severe consequence of unrecognized or inadequately managed acute compartment syndrome (ACS). It primarily affects the deep flexor compartments of the forearm, resulting in permanent flexion deformities due to ischemic muscle necrosis and subsequent fibrosis [1].

Although its pathophysiology is well established, evolving global health challenges such as COVID-19 may alter the typical disease course. Recent studies have highlighted that SARS-CoV-2 infection has been associated with systemic endothelial dysfunction, cytokine-driven inflammation, and immune-mediated neuropathies, which together can impair tissue perfusion and wound healing [2-10]. These mechanisms, combined with pandemic-related diagnostic delays and isolation protocols, may obscure or exacerbate early manifestations of compartment syndrome [2,3].

We present the case of a 55-year-old man with a delayed diagnosis of VIC following a mild episode of COVID-19. This case highlights how systemic inflammation, microvascular dysfunction, and care delays during the pandemic may have contributed to the evolution of ischemic soft-tissue injury.

## Case presentation

**Figure 1:**
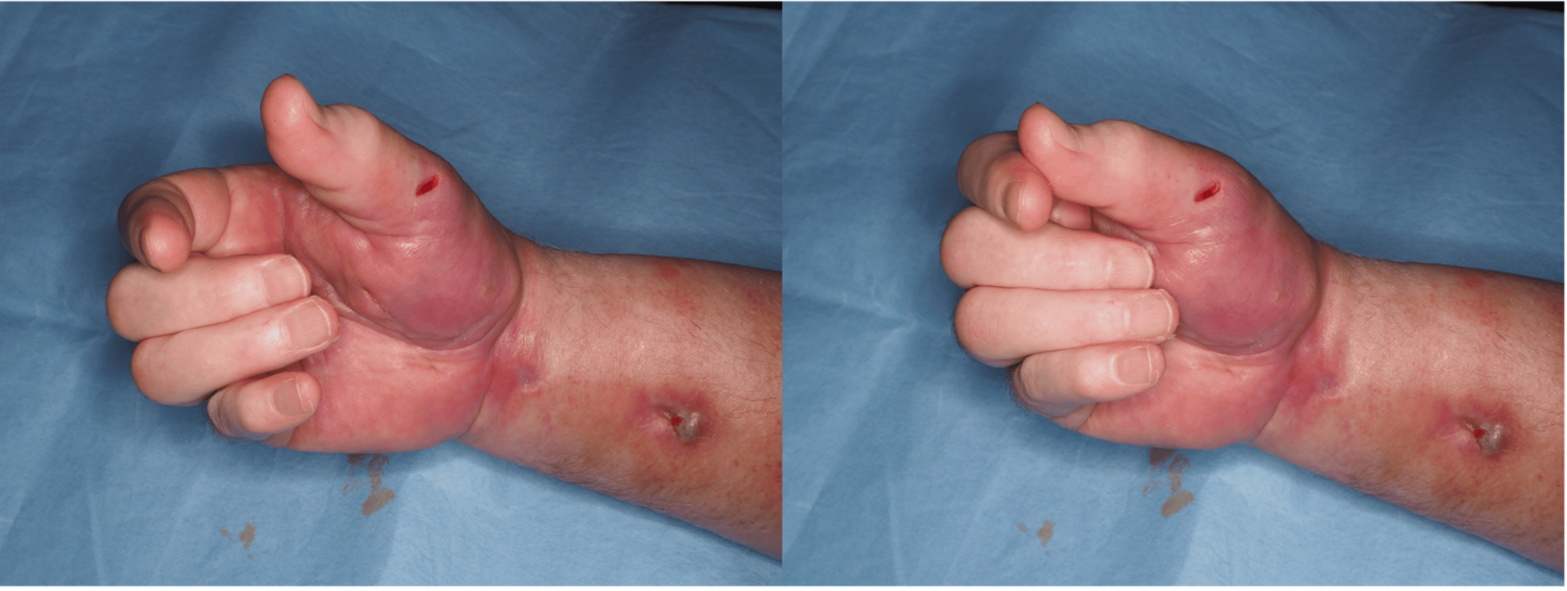
Preoperative view showing fixed flexion deformity of digits II–V

A 55-year-old man with a history of type 2 diabetes mellitus and an occupation involving manual labor presented with progressive flexion deformity and functional impairment of the right hand (Figure 1). There was no reported history of major trauma, prior forearm surgery, or known systemic vasculopathy. Six months before presentation, the patient experienced intermittent paresthesia and nocturnal numbness in the right hand, suggestive of a possible evolving compression neuropathy. During recovery from a mild COVID-19 infection managed at home, the patient developed acute swelling, pain, and stiffness of the right forearm.

After several days, the patient presented to a local hospital, where cellulitis was diagnosed by the emergency department based on forearm swelling, erythema, and tenderness. Multiple superficial incisions for presumed abscess drainage were made, though no purulence was found, and no tissue or fluid cultures were obtained. No imaging studies (X-ray, MRI, or angiography) were performed at that time. The patient received empirical antibiotic therapy, but no records of specific agents or dosing were available.

Despite outpatient wound care, empirical antibiotic therapy, and adjunctive ozone treatment, the forearm wounds failed to heal and progressed to chronic draining fistulae. No inflammatory laboratory data were available from the referring facility. Upon referral to our tertiary center several weeks later, the patient exhibited fixed flexion deformities of digits II-V, sensory loss in the median nerve distribution, volar forearm induration, and multiple non-healing fistulae along previous incision sites. 

A diagnosis of chronic Volkmann ischemic contracture was made based on clinical findings and disease chronology.

Surgical management

**Figure 2:**
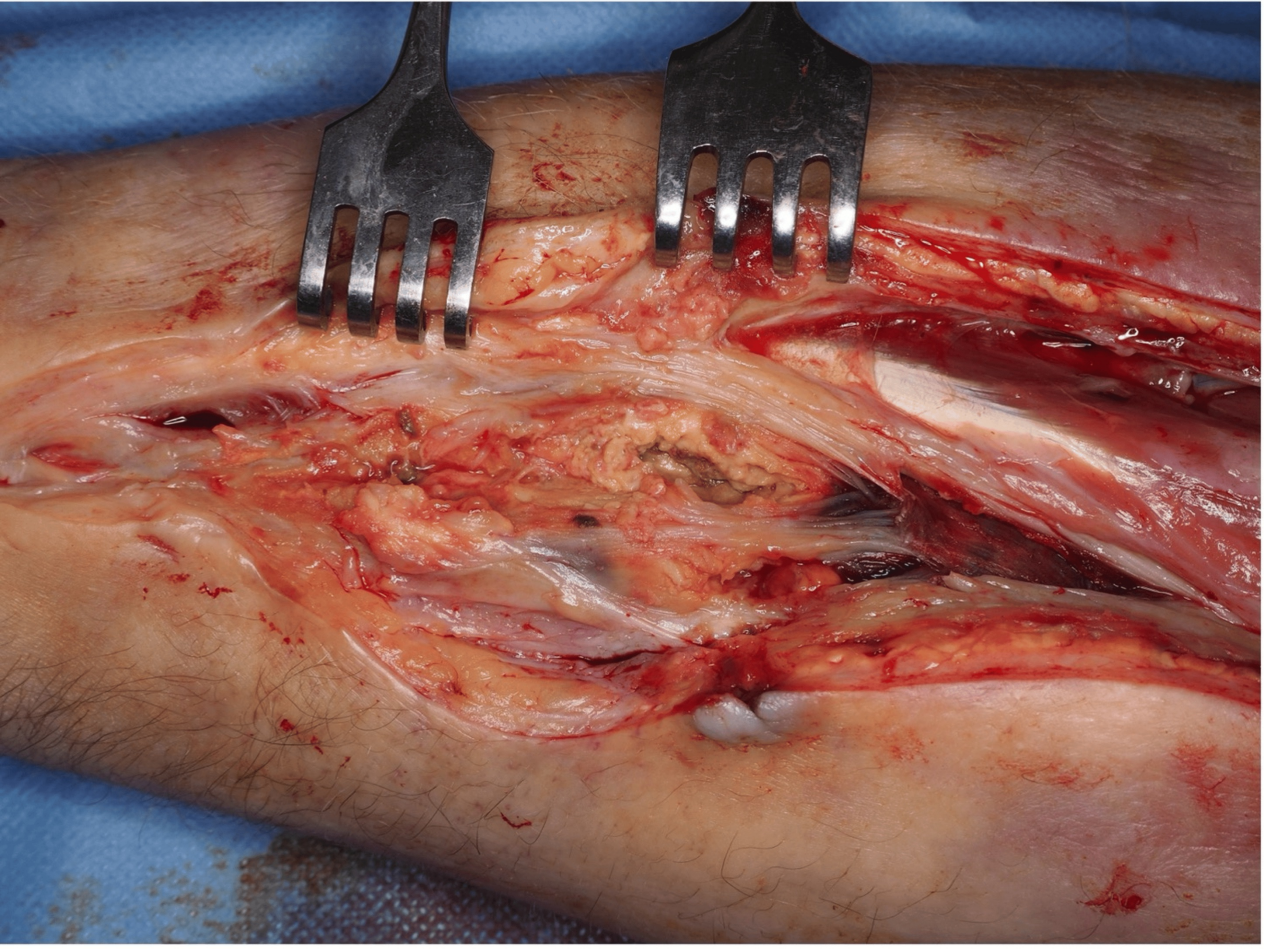
A multi-chambered necrotic area in the volar forearm was revealed during debridement

**Figure 3:**
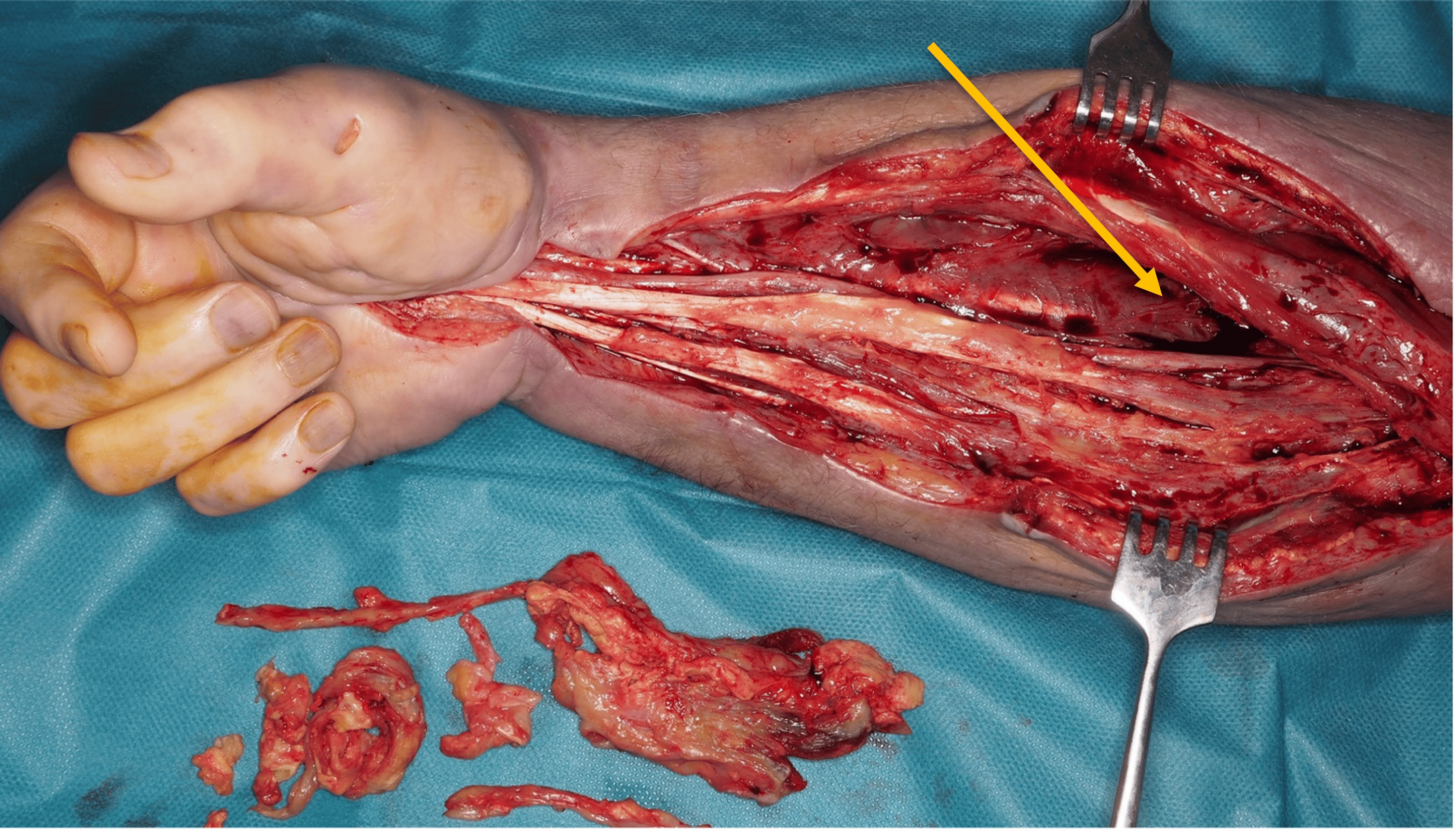
Intraoperative exposure of the median nerve (arrow) during neurolysis

The patient underwent surgical exploration through a volar S-shaped incision extending from the distal forearm to the antecubital fossa. Intraoperative findings revealed extensive fibrosis, multiple chronic fistula tracts, and areas of non-viable muscle consistent with chronic ischemia (Figure 2). The median nerve was identified and carefully neurolyzed from the carpal tunnel proximally to the antecubital region (Figure 3). Although the flexor tendons were structurally intact, they were densely adherent within the fibrotic bed. Thorough debridement of all necrotic and non-viable soft tissues was performed, with care taken to preserve functional structures where possible.

A flexor origin slide procedure was considered but deferred due to extensive fibrosis, poor muscle quality, and concern for vascular morbidity. Instead, limited debridement and cautious release were performed to decompress neurovascular structures, reduce tension, and improve mobility. The wound was closed using tension-reducing techniques to accommodate residual soft tissue deficits and prevent further ischemic compromise.

Early postoperative outcomes were favorable, with marked reduction in forearm pain, resolution of paresthesia, and progressive improvement in finger range of motion. The patient was enrolled in a structured hand therapy program with gradual mobilization and passive stretching.

Follow-up and outcomes

Postoperative recovery was notable for immediate pain relief and resolution of tightness. Within weeks, the patient regained partial motion in digits II-V and improved wrist rotation. Range of motion (ROM) assessment demonstrated partial active extension and flexion of the fingers, with progressive improvement in both active and passive mobility during rehabilitation. Pronation and supination were mildly restricted due to fibrosis but functionally sufficient.

**Figure 4:**
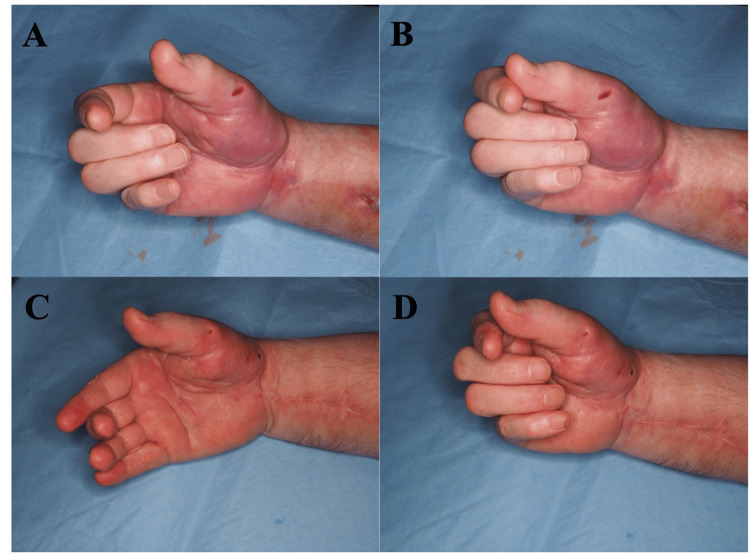
Hand function before and after surgery: (A and B) preoperative extension and flexion; (C and D) extension and flexion at three months postoperatively

At one-month follow-up, both active and passive motion had significantly improved (Figures 4A-4D). The patient reported restored ability to perform basic daily activities such as writing and gripping small objects. Ongoing physiotherapy and occupational therapy were continued, with incremental functional gains over three months.

Residual mild contracture of the proximal flexor group persisted. The patient remains under follow-up, and additional reconstructive procedures such as tenolysis or myolysis are under consideration for further functional improvement.

## Discussion

VIC represents the final, fibrotic stage of an untreated or inadequately managed ACS. First described by Richard von Volkmann in 1881, VIC was initially recognized as a complication of tight bandaging or trauma to the forearm resulting in muscle necrosis and contracture [1]. Despite advances in diagnosis and treatment, it remains a rare but serious and preventable condition, most commonly resulting from delayed recognition of compartment syndrome.

The differential diagnosis of VIC includes several conditions that may also lead to hand flexion deformities. Dupuytren’s contracture, for instance, presents as painless progressive digital flexion but is a fibromatosis of the palmar fascia, not related to ischemia. Similarly, stroke-related spastic contractures and chronic nerve entrapments (e.g., cubital or carpal tunnel syndrome) may mimic some motor findings of VIC but lack the hallmark signs of muscle necrosis and volar forearm fibrosis. Accurate history-taking and recognition of ischemic etiology are therefore critical for differentiation.

It is also important to distinguish between ACS and VIC. ACS is an acute surgical emergency marked by elevated intracompartmental pressure that compromises perfusion, resulting in ischemic injury if untreated. Classic signs include disproportionate pain, tense swelling, and pain on passive stretch. Timely fasciotomy can prevent permanent damage [6]. In contrast, VIC reflects the chronic fibrotic stage, presenting with fixed flexion deformities, neurosensory deficits, and volar contractures, features that typically require reconstructive rather than decompressive procedures [1,6].

In this case, the diagnosis of cellulitis at the initial presentation led to inappropriate incision and drainage procedures. No inflammatory laboratory data were available from the referring facility, and no purulence was encountered intraoperatively. Tissue or fluid cultures were not obtained, and no imaging was performed before intervention. These management choices likely contributed to the delayed recognition of the underlying ischemic process. While empirical antibiotic therapy was administered, the persistence of non-healing wounds and lack of clinical improvement retrospectively suggested a non-infectious etiology. The absence of microbiological data and early imaging represents a limitation of this case, and it highlights the need for thorough infection evaluation when clinical findings are ambiguous.

At presentation to our center, the diagnosis of chronic VIC was made clinically based on the presence of fixed digital flexion, median nerve sensory loss, and dense volar fibrosis. Physical examination showed severely restricted active and passive motion of digits II-V, mild limitation of pronation and supination, and increased flexor tightness upon wrist extension, findings typical of Volkmann contracture. Although radiological imaging, such as MRI or angiography, would have been valuable for assessing muscle viability and vascular status, these were not available due to resource constraints and the chronic stage of presentation. The absence of imaging is acknowledged as a limitation, but it did not preclude diagnosis, as intraoperative findings confirmed extensive fibrotic remodeling rather than acute inflammation.

Surgical management in this case focused on selective decompression and neurolysis rather than aggressive reconstruction. A flexor origin slide was considered but deferred due to extensive fibrosis, poor muscle quality, and the high risk of vascular compromise in a chronically ischemic limb. Limited debridement and cautious release were therefore performed to decompress neurovascular structures and remove non-viable tissue while preserving residual perfusion.

Although classical surgical guidance advises against fasciotomy beyond approximately 36 hours of untreated ischemia, given the likelihood of irreversible muscle necrosis and the risk of reperfusion injury, late surgical exploration may still be justified when the predominant pathology is chronic fibrosis and nerve entrapment rather than acutely ischemic but viable muscle [11,12]. In such cases, the goal of intervention is not to salvage threatened muscle but to relieve compression, release adhesions, and reduce functional impairment. This individualized approach aligns with contemporary recommendations that emphasize tailoring operative strategy to tissue viability and chronicity of injury [11,12].

Postoperative recovery relied on guided rehabilitation, with progressive work on ROM and functional reintegration. ROM was used as the primary outcome measure; no standardized scoring instruments were applied. Future cases would benefit from incorporating validated functional assessments to allow clearer comparison across reports. Despite the chronic stage of presentation, the patient experienced meaningful pain relief and partial restoration of hand mobility, indicating that selective late intervention can still provide symptomatic and functional improvement.

This case highlights how a combination of systemic illness, misdiagnosis, and underlying risk factors can culminate in VIC. Despite the absence of trauma, the patient’s clinical course, beginning with COVID-19-related forearm swelling misdiagnosed as cellulitis, progressed through unnecessary incisions, delayed treatment, and ultimately tissue necrosis and fibrosis. We propose five interrelated mechanisms through which COVID-19 may have contributed to the pathogenesis of VIC.

COVID-19-induced exacerbation of pre-existing neuropathy

The patient had prodromal symptoms consistent with early pronator syndrome several months before deterioration. SARS-CoV-2 has been shown to exacerbate peripheral neuropathies through immune-mediated and direct viral mechanisms [8,10]. These include molecular mimicry, autoantibody formation (e.g., anti-ganglioside antibodies), and disruption of the blood-nerve barrier by cytokines such as IL-6 and TNF-α [10]. Such mechanisms may have amplified a subclinical compressive neuropathy into a fulminant ischemic insult.

Hyperinflammatory state and cytokine-driven edema

COVID-19 is characterized by a systemic inflammatory response with elevated cytokines, including IL-6, IL-1β, TNF-α, and interferon-γ [3]. This can increase vascular permeability and lead to capillary leak, resulting in interstitial edema. In confined compartments like the volar forearm, this may raise pressure beyond perfusion thresholds, causing ACS [3,4,7]. Musculoskeletal symptoms reported in long COVID, such as myalgia, fatigue, and weakness, support the presence of persistent inflammatory effects on soft tissues even post-infection [3].

Endothelial dysfunction and capillary leak syndrome

Endothelial damage, now recognized as central to COVID-19 pathophysiology, promotes microvascular injury, capillary leak, and ischemia [2,5,9]. Mechanisms include nitric oxide depletion, cytokine-induced endotheliitis, and direct viral invasion of endothelial cells [9]. These could plausibly contribute to compartment pressure elevation and impaired perfusion, as observed in our patient.

Diagnostic delay and misinterpretation due to COVID-19 context

The patient experienced a delay in seeking medical attention due to isolation protocols and was misdiagnosed with cellulitis of the forearm. Multiple superficial incisions were performed without finding abscesses. Such misinterpretation is not uncommon, as early ACS can mimic infection with signs like swelling and erythema [13]. Together, these factors allowed the condition to progress from reversible ischemia to irreversible contracture [7,14].

Impaired wound healing and superinfection

Following inappropriate surgery, the patient developed draining fistulae and persistent inflammation. COVID-19 is known to impair all phases of wound healing through downregulation of ACE2, sustained cytokine activity (IL-6, TNF-α, IFN-γ), oxidative stress, and depletion of epidermal stem cells [15]. Moreover, neutrophil heterogeneity and macrophage dysfunction in COVID-19 prolong inflammation and reduce tissue repair capacity [16]. These mechanisms likely contributed to poor wound healing, despite localized disease and multiple interventions [16].

Although most cases of COVID-associated compartment syndrome have been reported in critically ill patients, often with high mortality rates up to 43%, our case illustrates that significant functional recovery remains possible in non-severe cases if surgical management is timely and rehabilitation is initiated early [14].

This case report is limited by the absence of radiological imaging, tissue cultures, and laboratory inflammatory markers, all of which would have strengthened the diagnostic evaluation by helping to differentiate infection from ischemia and delineate the extent of tissue injury. Although intraoperative findings and clinical features were consistent with chronic ischemic fibrosis rather than active infection, the lack of these diagnostic modalities prevents definitive exclusion of alternative etiologies. As with all single-case reports, causality between COVID-19 and VIC cannot be established; the proposed mechanisms should therefore be interpreted as plausible contributors rather than confirmed etiological factors. Similar reports describing COVID-19-associated limb ischemia, viral myositis, and compartment syndrome in both critically ill and non-critical patients suggest that SARS-CoV-2-related endothelial dysfunction and inflammatory dysregulation may play a role in ischemic soft-tissue injury [5,7-10]. However, these associations remain speculative. The limitations observed in this case underscore the diagnostic value of MRI, angiography, and microbiological sampling in future cases and highlight the need for systematic data collection and further research to clarify whether COVID-19 contributes to the development or progression of compartment syndrome and to identify which patients may be most susceptible.

## Conclusions

VIC remains a preventable but serious consequence of missed ACS. This case illustrates how systemic factors such as COVID-19, diabetes, and neuropathy may obscure early diagnosis and allow ischemic injury to progress. COVID-19 should be viewed as a potential contributing factor rather than a definitive cause, given its known inflammatory and vascular effects. Heightened clinical suspicion, prompt differentiation from soft-tissue infection, and timely surgical intervention are essential to prevent irreversible functional impairment. Even when diagnosis is delayed, decompression and neurolysis combined with structured rehabilitation can yield meaningful recovery. Further systematic data collection and dedicated research cohorts are needed to clarify whether SARS-CoV-2 contributes to ischemic musculoskeletal complications and to identify patients who may be at increased risk.
